# Real‑world analysis of Macular Oedema associated with Paclitaxel Formulations using the Japanese Adverse Drug Event Report database

**DOI:** 10.1371/journal.pone.0354959

**Published:** 2026-07-29

**Authors:** Koki Takeda, Toshinori Hirai, Ayu Sugioka, Rinka Okamura, Asako Nishimura, Nobuhito Shibata

**Affiliations:** 1 Laboratory of Medical Pharmaceutics, Faculty of Pharmaceutical Sciences, Doshisha Women’s College of Liberal Arts, Kyotanabe, Kyoto, Japan; 2 Department of Pharmacy, Institute of Science Tokyo Hospital, Bunkyo-ku, Tokyo, Japan; 3 Education and Research Center for Clinical Pharmacy, Faculty of Pharmacy, Osaka Medical and Pharmaceutical University, Takatsuki, Osaka, Japan; Guangdong Nephrotic Drug Engineering Technology Research Center, Institute of Consun Co. for Chinese Medicine in Kidney Diseases, CHINA

## Abstract

**Aim:**

This study explored the potential association between paclitaxel (PTX) and macular oedema (ME), a rare adverse event, through pharmacovigilance analysis using a large-scale spontaneous reporting database.

**Methods:**

Disproportionality analysis of ME associated with PTX formulations, including nanoparticle albumin-bound PTX (nab‑PTX) was conducted using the Japanese Adverse Drug Event Report database on data collected during April 2004–June 2025. Multivariable logistic regression analysis was used to estimate adjusted reporting odds ratios (RORs) for ME. A positive signal was defined as a lower 95% confidence interval (CI) for the ROR exceeding 1.0 with at least three ME cases.

**Results:**

Among 585,738 reports, 123 involved ME. A significant signal was observed for overall PTX (adjusted ROR: 19.5; 95% CI: 13.5–28.3). By formulation, significant signals were identified for conventional PTX (adjusted ROR: 9.4; 95% CI: 5.5–16.1) and nab‑PTX (adjusted ROR: 47.0; 95% CI: 30.4–72.8). Multivariable logistic regression indicated significant signals for diabetes mellitus (adjusted ROR: 1.8; 95% CI: 1.2–2.9) and co-administration of prostaglandin analogue eye drops (adjusted ROR: 19.9; 95% CI: 8.6–45.7).

**Conclusion:**

PTX formulations were associated with an increased reporting signal of ME. Notably, patients with diabetes mellitus and those using prostaglandin analogue eye drops may require closer ophthalmologic monitoring during PTX therapy.

## Introduction

Macular oedema (ME) involves swelling of part of the retina, the light‑sensitive tissue at the back of the eye [1], which can cause visual impairment and markedly reduce quality of life, potentially leading to irreversible blindness in critical cases [2]. The pathophysiology of ME is multi-factorial, involving diabetes mellitus, age‑related macular degeneration, retinitis pigmentosa, uveitis, retinal vein occlusion, ocular surgery and certain medications, including paclitaxel (PTX) [3–9]. Reports of drug‑induced ME are increasing; it can occur unpredictably and is often overlooked in clinical practice [10]. PTX has been reported to induce ME; it binds to tubulin, promoting microtubule polymerisation, which can disrupt retinal pigment epithelial function and cause severe ocular damage [11].

PTX serves as a primary cytotoxic compound integral to established chemotherapy protocols for non-small cell lung cancer (NSCLS) and ovarian cancer [12,13], whereas nanoparticle albumin-bound paclitaxel (nab‑PTX) is used as a core chemotherapy regimen for NSCLS and pancreatic cancer [12,14]. However, evidence of PTX‑induced ME from large‑scale analyses remains limited, with only small case reports published [15,16]. ME is a rare adverse event, reported in 0.1%–0.4% of cases [17], making evaluation of PTX-associated ME in clinical trials challenging and restricting efforts to prevent ME in clinical practice.

The Japanese Adverse Drug Event Report (JADER) database is a spontaneous adverse drug reaction reporting system with extensive and diverse data, allowing hypothesis generation on risk factors for PTX-associated ME. In the present pharmacovigilance study, signal detection analysis of PTX-associated ME was conducted using this database. The analysis aimed to identify potential risk factors and generate hypotheses to guide clinical monitoring and mitigation strategies for ME cases.

## Materials and methods

### Study population and data collection

**Fig 1:**
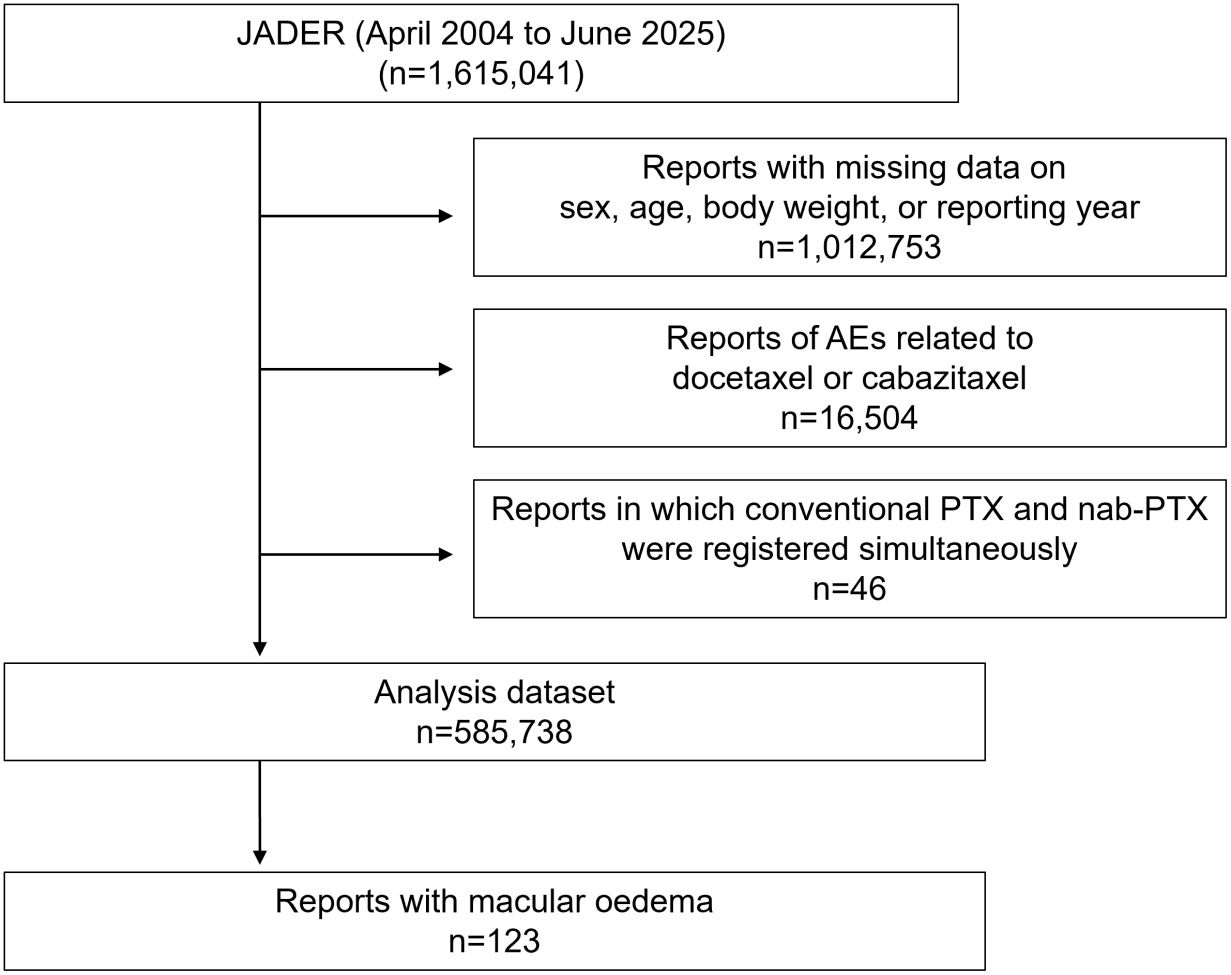
Flowchart of patient selection. Analysed dataset comprised 585,738 reports after excluding (1) records with missing data (n = 1,012,753), (2) reports involving docetaxel or cabazitaxel (n = 16,504) and (3) reports with simultaneous registration of PTX and nab-PTX (n = 46) from the original 1,615,041 reports. In total, 123 cases of ME were identified.

Adverse event (AE) data were obtained from the JADER database on the Pharmaceuticals and Medical Devices Agency (PMDA) website (http://www.pmda.go.jp/), which is fully anonymised by the PMDA. Individual informed consent was not required as JADER is a publicly accessible database. The JADER database comprised 1,615,041 spontaneous reports integrating demographic information (DEMO), drug administration information, comorbidity information and AE information collected during April 2004–June 2025 (Fig 1). The analysed dataset was obtained after applying the following exclusion criteria: (1) reports with missing data on sex, age, body weight or reporting year; (2) reports of AEs associated with docetaxel or cabazitaxel; and (3) reports where conventional PTX and nab‑PTX were both registered. Data were pre-screened to remove duplicates and incomplete entries. In the DEMO dataset, age was categorised in 10-year increments, whereas body weight was recorded in 10‑kg intervals.

### AE detection

The Japanese version of the Medical Dictionary for Regulatory Activities (MedDRA/J v28.1) was employed as the standardised terminology for coding and analysing AE data. ME events were defined using the preferred term codes for ‘macular oedema’ [10025415] and ‘cystoid macular oedema’ [10058202]. Extracted variables included sex, age, body weight, reporting year, co-existing diabetes mellitus, concomitant prostaglandin analogue eye drop use and ME occurrence. Co-existing diabetes mellitus was specifically defined using preferred terms in the Standardised MedDRA Query for both ‘hyperglycaemia’ and ‘new onset diabetes mellitus’ [20000041].

### Statistical analysis

The signal for ME occurrence was evaluated using disproportionality analysis with a two-by-two contingency table for PTX formulations (conventional PTX and nab-PTX). Reporting odds ratios (RORs) and corresponding 95% confidence intervals (CIs) were calculated according to equations (1) and (2):


$$\begin{array}{c} \;ROR=\frac{\left(\frac{a}{c}\right)}{\left(\frac{b}{d}\right)}=\frac{ad}{bc}\; \end{array}$$ (1)

$$\begin{array}{c} \text{9}\text{5}\%\;CI=exp\;\left\{log(ROR)\;\pm \;\text{1}.\text{9}\text{6}\;\sqrt{\frac{\text{1}}{a}+\frac{\text{1}}{b}+\frac{\text{1}}{c}+\frac{\text{1}}{d}}\right\} \end{array}$$ (2)

where *a* represents patients receiving the target drug with ME, *b* represents those not receiving the target drug with ME, *c* represents those receiving the target drug without ME and *d* represents those not receiving the target drug without ME. A positive signal was defined as a lower 95% CI for the ROR exceeding 1.0 with a minimum of three ME cases [18].

Multivariable logistic regression analysis was performed to calculate adjusted RORs of ME for PTX formulations, incorporating covariates including sex, age (≥70 years), body weight (<50 kg), co-existing diabetes mellitus and co-administration of prostaglandin analogue eye drops. Covariates were selected based on previous studies [3,19–21]. All statistical analyses were performed using R (v4.5.2), with *P* < 0.05 considered statistically significant.

## Results

### Study population

Fig 1 presents a flowchart summarising the selection of the study population. The JADER dataset initially comprised 1,615,041 reports. After applying the following exclusion criteria, 585,738 reports were retained: (1) missing data on sex, age, body weight and reporting year (n = 1,012,753); (2) use of docetaxel or cabazitaxel (n = 16,504); and (3) simultaneous registration of conventional PTX and nab-PTX (n = 46). In the final dataset, 123 reports of ME were identified.

Table 1 summarises the clinical characteristics of the study population. Among the reports, 308,573 (52.68%) were for male patients, 246,824 (42.14%) involved patients aged ≥70 years and 203,737 (34.78%) involved patients weighing <50 kg. Reports for patients receiving the study drugs totalled 16,192 (2.76%), including 11,563 (1.97%) with conventional PTX and 4,629 (0.79%) with nab‑PTX. Co-existing diabetes mellitus was reported in 79,493 cases (13.57%) and 1,770 reports (0.30%) involved co-administered prostaglandin analogue eye drops.

**Table 1:** Clinical characteristics in the analysis dataset.

Variable	n = 585,738
Sex	
Male	308573 (52.68)
Female	277165 (47.32)
Age, years	
<10	25166 (4.30)
10–19	16804 (2.87)
20–29	17193 (2.94)
30–39	27475 (4.69)
40–49	42016 (7.17)
50–59	74331 (12.69)
60–69	135929 (23.21)
70–79	161449 (27.56)
80–89	75189 (12.84)
90–99	10042 (1.71)
>100	144 (0.02)
Body weight, kg	
<10	12082 (2.06)
10–19	8616 (1.47)
20–29	7090 (1.21)
30–39	39352 (6.72)
40–49	136597 (23.32)
50–59	179654 (30.67)
60–69	123361 (21.06)
70–79	51873 (8.86)
80–89	17000 (2.90)
90–99	5759 (0.98)
100–109	2071 (0.35)
110–119	849 (0.14)
120–129	334 (0.06)
130–139	147 (0.03)
140–149	187 (0.03)
150–159	346 (0.06)
160–169	227 (0.04)
170–179	98 (0.02)
180–189	20 (0.00)
190–199	2 (0.00)
>200	73 (0.00)
Diabetes mellitus	79493 (13.57)
PTX formulations	16192 (2.76)
PTX	11563 (1.97)
nab-PTX	4629 (0.79)
Prostaglandin analogue eye drop	1770 (0.30)

Abbreviations: PTX, paclitaxel; nab-PTX, nab-paclitaxel.

Data are presented as numbers (%).

### Disproportionality analysis of PTX formulations for ME

Table 2 presents the disproportionality analysis results for PTX-associated ME. Multivariable logistic regression analysis revealed a positive signal for overall PTX formulations (adjusted ROR: 19.5; 95% CI: 13.5–28.3). By formulation, positive signals were observed for conventional PTX (adjusted ROR: 9.4; 95% CI: 5.5–16.1) and nab‑PTX (adjusted ROR: 47.0; 95% CI: 30.4–72.8).

**Table 2:** Reporting odds ratios for macular oedema associated with paclitaxel formulations.

	With ME	Without ME	ROR	95% CI	Adjusted ROR	95% CI
PTX formulations	44	16,148	19.6	13.6–28.4	19.5	13.5–28.3
PTX	16	11,547	10.0	5.8–17.1	9.4	5.5–16.1
nab-PTX	28	4,601	43.9	28.5–67.6	47.0	30.4–72.8
Reference	79	569,467	1	–	1	–

ME, macular oedema; ROR, reporting odds ratio; 95% CI, 95% confidence interval; PTX, paclitaxel; nab-PTX, nab-paclitaxel.

The number of patients with and without macular oedema in each category are presented.

A multivariable logistic regression model was used to determine the adjusted ROR by introducing sex, age (>70 years), body weight (<50 kg), diabetes mellitus, and co-administration of medications (paclitaxel/nab-paclitaxel and prostaglandin analogue eye drop).

Table 3 shows the multivariable logistic regression analysis results considering clinical characteristics. In addition to PTX use, positive signals were detected for co-existing diabetes mellitus (adjusted ROR: 1.8; 95% CI: 1.2–2.9) and co-administration of prostaglandin analogue eye drops (adjusted ROR: 19.9; 95% CI: 8.6–45.7).

**Table 3:** Multivariable logistic regression analysis for signals of macular oedema associated with paclitaxel formulations.

	With ME	Without ME	Adjusted ROR	95% CI
PTX formulations	44	16,148	19.5	13.5–28.3
Sex, male	55	308,518	0.8	0.5–1.1
Age ≥ 70 years	39	246,785	0.6	0.4–0.9
Body weight <50 kg	42	203,695	1.0	0.7–1.5
Diabetes mellitus	25	79,468	1.8	1.2–2.9
Prostaglandin analogue eye drop	6	1,764	19.9	8.6–45.7
Reference	79	569,467	1.0	–

ME, macular oedema; ROR, reporting odds ratio; 95% CI, 95% confidence interval; PTX, paclitaxel.

The number of patients with and without macular oedema in each category are presented.

A multivariable logistic regression model was used to determine the adjusted ROR by introducing sex, age (≥70 years), body weight (<50 kg), diabetes mellitus, and co-administration of medications (paclitaxel/nab-paclitaxel and prostaglandin analogue eye drop).

## Discussion

To our knowledge, this is the first study to identify a signal of ME associated with PTX formulations using the JADER database. PTX-associated ME appears more likely to occur in patients with diabetes mellitus and in those using prostaglandin analogue eye drops.

The pathogenesis of ME involves fluid accumulation within the outer plexiform and inner nuclear layers [22]. In diabetic ME, vascular endothelial growth factor signalling further compromises the blood–retinal barrier by promoting inflammatory pathways. Accordingly, diabetic ME responds to anti‑vascular endothelial growth factor (VEGF) inhibitors [23]. In contrast, PTX formulations may directly damage the retinal pigment epithelium and Müller cells, both essential for maintaining the blood–retinal barrier [24]. Therefore, early detection of PTX-associated ME is critical, as anti-VEGF inhibitors are ineffective in PTX-induced cases [16].

As shown in S1 Table, overall PTX formulations were the most frequently reported drugs associated with ME in the analysed dataset. Our analysis revealed positive signals for overall PTX as well as for each formulation. Although direct comparisons between PTX formulations are limited in this study, nab‑PTX has been reported to confer a higher risk of peripheral neuropathy compared with conventional PTX [24]. This difference is thought to reflect formulation characteristics, as conventional PTX is dissolved in polyoxyethylated castor oil, whereas nab‑PTX is bound to human serum albumin [17,25]. The increased peripheral neuropathy risk may relate to altered immune responses and tissue distribution. In a large-scale study of 14,260 patients treated with taxanes, ME incidence was significantly higher with nab‑PTX than with PTX (0.4% vs. 0.1%) [17]. Previous studies have also reported a dose-dependent pattern in PTX-associated ME [15]. In breast cancer, the body surface area–adjusted dose of both conventional PTX and nab-PTX is higher than that in other regimens, warranting particular caution [26,27]. Although definitive conclusions regarding comparative ME risk between PTX formulations cannot be drawn, clinicians should remain vigilant irrespective of formulation. In addition, the data on PTX dosage and treatment duration were unavailable in our analysis. Since the cumulative dosage of PTX has been reported to be associated with the development of ME, these variables may be required for further analysis aimed at the early detection of ME [28].

Among reports of PTX-induced ME, breast cancer was the most common malignancy (S2 Table). For cases receiving conventional PTX, carboplatin and bevacizumab were frequently co-administered. However, limited evidence exists regarding ME risk stratified by cancer types or concomitant anti-cancer therapy. Although several case reports have described ME during combination regimens, PTX has consistently been implicated rather than accompanying agents [29,30]. Conversely, pancreatic cancer accounted for the highest number of reports in the nab-PTX subgroup (S3 Table). Gemcitabine plus nab-PTX constitutes a cornerstone chemotherapy regimen for pancreatic cancer [31]. Prior studies have suggested that nab‑PTX possesses intrinsic retinal toxicity [32,33]. In the present dataset, limited case numbers precluded sensitivity analyses by cancer type or co‑administrated anti-cancer agents. Further large-scale analyses are needed to clarify ME risk across subgroups.

Multivariable analysis identified diabetes mellitus and prostaglandin analogue eye drops as additional positive signals for ME. Hyperglycaemia has been associated with increased chemotherapy‑related AEs and diabetes mellitus is known to exacerbate PTX‑induced peripheral neuropathy [34,35]. Moreover, diabetes mellitus causes systemic microvascular injury and may increase ME risk through overlapping mechanisms, including hyperglycaemia-induced oxidative stress, chronic inflammation and endothelial dysfunction [36,37]. Although it remains uncertain whether diabetes mellitus increases the incidence of medication-induced ME, our findings suggest that it may contribute to PTX-associated ME. A theoretical association between prostaglandin analogue eye drops and ME has been proposed, as prostaglandins increase vascular permeability and may disrupt the blood–retinal barrier, leading to macular fluid accumulation [38]. Several case series have described ME development following initiation of these eye drops in patients already receiving other glaucoma treatments [39]. These findings imply a potentially higher ME risk compared with alternative glaucoma therapies or a synergistic interaction with concomitant medications. In contrast, few reports have described ME following prostaglandins administered via non-ophthalmic routes [40]. Additionally, ME development has been linked to factors such as retinal disease, ocular surgery, aphakia or subluxated intraocular lens rather than prostaglandin analogue eye drops alone [9,21]. Thus, the administration route of prostaglandin may be relevant when evaluating PTX-associated ME.

This study has several limitations that affect interpretation. First, because JADER comprises spontaneous reports, reporting biases, including underreporting, notoriety bias, ripple effects and the Weber effect, are unavoidable [41]. Second, reports with missing data such as sex, age, body weight, and reporting year in JADER were excluded from the present analysis, resulting in the exclusion of more than one million reports, which may have introduced selection bias. Third, the database lacks information on patients receiving PTX without adverse effects, precluding incidence estimation. Fourth, dosage and detailed regimens were unavailable for analysis. Finally, reports involving prostaglandin analogue eye drops often co-existed with ocular diseases such as retinal disease and history of ocular surgery [9,21,38]. Therefore, prostaglandin analogue eye drops might be a confounding factor in the detection of signals for ME. These limitations underscore the need for prospective studies incorporating detailed clinical data, including dosage, treatment duration, ocular history and comprehensive ophthalmologic assessments, to better define the risk factors and mechanisms of PTX‑induced ME.

In conclusion, our results indicate that PTX formulations are associated with an increased reporting signal of ME. Furthermore, diabetes mellitus and prostaglandin analogue eye drops may further elevate this risk. However, early ophthalmic monitoring could facilitate timely detection of PTX-associated ME. Further large-scale studies are warranted to validate these findings and clarify underlying mechanisms.

## Supporting information

S1 Table Top 10 most frequently co‑administered drugs in the macular oedema reports. (DOCX)

S2 Table Reports of co-existing cancers, co-administered anti-cancer drugs and macular oedema in cases involving paclitaxel use. (DOCX)

S3 Table Reports of co-existing cancers, co-administered anti-cancer drugs and macular oedema in cases involving nab-paclitaxel use. (DOCX)
