# Supplementary material for: Real‑world analysis of Macular Oedema associated with Paclitaxel Formulations using the Japanese Adverse Drug Event Report database
Source: PLoS One. 2026 Jul 29;21(7):e0354959. doi: 10.1371/journal.pone.0354959 (PMC13419171; doi:10.1371/journal.pone.0354959)
Supplement: S1 Table — (DOCX) [file pone.0354959.s001.docx]

**Supplementary Table 1. Top 10 most frequently co‑administered drugs in the macular oedema reports**

| Drug | count |
| --- | --- |
| Paclitaxel formulations | 44 |
| Gemcitabine Hydrochloride | 19 |
| Fingolimod Hydrochloride | 14 |
| Dexamethasone Sodium Phosphate | 12 |
| Encorafenib | 11 |
| Binimetinib | 11 |
| Famotidine | 11 |
| Loxoprofen Sodium Hydrate | 11 |
| Magnesium Oxide | 10 |
| Cetuximab | 9 |
