# Supplementary material for: Real‑world analysis of Macular Oedema associated with Paclitaxel Formulations using the Japanese Adverse Drug Event Report database
Source: PLoS One. 2026 Jul 29;21(7):e0354959. doi: 10.1371/journal.pone.0354959 (PMC13419171; doi:10.1371/journal.pone.0354959)
Supplement: S2 Table — (DOCX) [file pone.0354959.s002.docx]

**Supplementary Table 2. Reports of co-existing cancers, co-administered anti-cancer drugs and macular oedema in cases involving paclitaxel use**

|  |  | With ME | Without ME |
| --- | --- | --- | --- |
| Co-existing cancers |  |  |  |
|  | Breast cancer | 7 | 1945 |
|  | Cervical cancer | 0 | 463 |
|  | Oesophageal cancer | 0 | 131 |
|  | Head and cheek cancer | 0 | 71 |
|  | NSCLC | 1 | 1740 |
|  | Ovarian cancer | 2 | 1299 |
|  | Stomach cancer | 0 | 1221 |
| Co-administered anti-cancer drugs |  |  |  |
|  | Atezolizumab | 0 | 833 |
|  | Bevacizumab | 4 | 3011 |
|  | Carboplatin | 4 | 6329 |
|  | Cetuximab | 1 | 251 |
|  | Cisplatin | 0 | 494 |
|  | Cyclophosphamide | 0 | 313 |
|  | Doxorubicin | 0 | 226 |
|  | Ipilimumab | 0 | 1052 |
|  | Nivolumab | 0 | 1437 |
|  | Pertuzumab | 1 | 117 |
|  | Ramucirumab | 0 | 1040 |
|  | Trastuzumab | 2 | 481 |

Abbreviations: ME, macular oedema; NSCLC, non-small cell lung cancer.
