# Supplementary material for: Real‑world analysis of Macular Oedema associated with Paclitaxel Formulations using the Japanese Adverse Drug Event Report database
Source: PLoS One. 2026 Jul 29;21(7):e0354959. doi: 10.1371/journal.pone.0354959 (PMC13419171; doi:10.1371/journal.pone.0354959)
Supplement: S3 Table — (DOCX) [file pone.0354959.s003.docx]

**Supplementary Table 3. Reports of co-existing cancers, co-administered anti-cancer drugs and macular oedema in cases involving nab-paclitaxel use**

|  |  | With ME | Without ME |
| --- | --- | --- | --- |
| Co-existing cancers |  |  |  |
|  | Breast cancer | 5 | 768 |
|  | NSCLC | 0 | 1094 |
|  | Pancreatic cancer | 17 | 1012 |
|  | Stomach cancer | 3 | 437 |
| Co-administration anti-cancer drugs |  |  |  |
|  | Atezolizumab | 2 | 606 |
|  | Carboplatin | 0 | 1868 |
|  | Gemcitabine | 17 | 1194 |
|  | Pembrolizumab | 0 | 597 |
|  | Ramucirumab | 3 | 399 |

ME, macular oedema; NSCLC, non-small cell lung cancer.
