# Supplementary material for: Real‑world analysis of Macular Oedema associated with Paclitaxel Formulations using the Japanese Adverse Drug Event Report database
Source: PLoS One. 2026 Jul 29;21(7):e0354959. doi: 10.1371/journal.pone.0354959 (PMC13419171; doi:10.1371/journal.pone.0354959)
Supplement: Attachment — Submitted filename: PLOS ONE_review_revise.ver.3.docx [file pone.0354959.s004.docx]

May 19, 2026

PLOS ONE

Academic Editor: Junzheng Yang, Ph.D.

Dear Editors,

We are grateful to the reviewers for their valuable and kind comments regarding our manuscript entitled “Real‑world analysis of macular oedema associated with paclitaxel formulations using the Japanese Adverse Drug Event Report database (PONE-D-26-18562).” We have revised the manuscript in accordance with the reviewers' suggestions and comments. Our revisions are highlighted in yellow in the revised manuscript. Our point-by-point responses to these comments are provided below. We have made some additional revisions during the proofreading process that we considered necessary. These sentences and words are also highlighted in yellow in the revised manuscript.

We believe that the revised manuscript has been significantly improved and hope that you now find it suitable for publication in the PLOS ONE.

We look forward to hearing the Editors’ decision on our manuscript.

Sincerely,

Koki Takeda, Ph.D.

Laboratory of Medical Pharmaceutics, Faculty of Pharmaceutical Sciences, Doshisha Women’s College of Liberal Arts, Kyotanabe, Kyoto 610-0332, Japan.

Phone: +81-77-465-8628

E-mail: k-takeda@dwc.doshisha.ac.jp

**Responses to the comments by Reviewer 1:**

This is a well-conducted and clinically relevant pharmacovigilance study addressing an uncommon but potentially vision-threatening adverse effect of paclitaxel therapy. The manuscript is interesting to read, clearly written, and tackles an important question with practical implications for oncologists, ophthalmologists, and pharmacists. The use of the Japanese Adverse Drug Event Report database provides a valuable real-world perspective, and the finding of stronger reporting signals with nab-paclitaxel, as well as the identification of diabetes mellitus and prostaglandin analogue eye drops as potential risk factors, adds useful clinical insight.

The study is generally well structured, with appropriate statistical methods and a balanced discussion of the inherent limitations of spontaneous reporting databases. I particularly appreciated that the authors avoided overstating causality and appropriately framed the results as signal detection and hypothesis generation.

**Response**: We sincerely appreciate your favorable assessment of the clinical significance of our study and the overall validity of our analyses. Your thoughtful evaluation is highly encouraging. We hope that the findings of this study will contribute to the managing the early detection of paclitaxel-associated macular oedema.

1) First, the rationale for excluding more than one million reports with missing covariate data should be discussed in greater detail, as this may introduce selection bias.

**Response**: The reviewer 1 raised an important point. As you pointed out, the possibility of selection bias due to missing data can affect findings in this study. Therefore, we have added a description in the Discussion.

P. 12, L. 189 (Discussion)

Second, reports with missing data such as sex, age, body weight, and reporting year in JADER were excluded from the present analysis, resulting in the exclusion of more than one million reports, which may have introduced selection bias.

2) Second, although the discussion mentions the possible influence of dose and cancer type, it would be helpful to clarify more explicitly how the lack of dosage, treatment duration, and ophthalmic baseline data may have affected the interpretation of the observed signals.

**Response**: We wish to express your appreciation for valuable comment. As you pointed out, cumulative dosage has been identified as a risk factor for paclitaxel-related macular oedema in the latest retrospective cohort study. In contrast, our dataset lacked data on dosage and treatment duration, therefore, we regarded this point as a potential limitation when conducting signal detection. Furthermore, ophthalmic backgrounds such as retinal disease, ocular surgery, aphakia or subluxated intraocular lens also have been reported to be risk factors for macular oedema, which influence our findings based on the following comment. We have added the description and references in the discussion section.

P. 10, L. 154 (Discussion)

In addition, the data on PTX dosage and treatment duration were unavailable in our analysis. Since the cumulative dosage of PTX has been reported to be associated with the development of ME, these variables may be required for further analysis aimed at the early detection of ME [28].

28. Sunjin H, Jiyeong K, Jee-Eun C, Seong J A, Maculopathy in Paclitaxel Users: Nationwide Drug Use, Incidence, and Risk Factors. Ophthalmol Retina. 2026: S2468-6530(26)00123-5. doi: 10.1016/j.oret.2026.03.019.

P. 12, L. 183 (Discussion)

Additionally, ME development has been linked to factors such as retinal disease, ocular surgery, aphakia or subluxated intraocular lens rather than prostaglandin analogue eye drops alone. Thus, the administration route of prostaglandin may be relevant when evaluating PTX-associated ME.

P. 12, L. 193 (Discussion)

Finally, reports involving prostaglandin analogue eye drops often co-existed with ocular diseases such as retinal disease and history of ocular surgery [21,38,41]. Therefore, prostaglandin analogue eye drops might be a confounding factor in the detection of signals for ME.

21. Muayad J, Mukhtar AO, Bordbar DD, Loya A, Alsoudi AF, Weng CY, et al. A large-scale cohort analysis of topical prostaglandin analog use and pseudophakic cystoid macular edema following cataract surgery. Ophthalmol Glaucoma. 2026;25: 276-281. doi: 10.1016/j.ogla.2025.12.010.

38. Moroi SE, Gottfredsdottir MS, Schteingart MT, Elner SG, Lee CM, Schertzer RM, et al. Cystoid macular edema associated with latanoprost therapy in a case series of patients with glaucoma and ocular hypertension. Ophthalmology. 1999;106: 1024-1029. doi: 10.1016/S0161-6420(99)00528-X.

41. Zhou Y, Bicket AK, Marwah S, Stein JD, Kishor KS, Pershing S, et al. Incidence of acute cystoid macular edema after starting a prostaglandin analog compared with other classes of glaucoma medications. Ophthalmol Glaucoma. 2025;8: 4-11.

3) Third, because prostaglandin analogue eye drops may themselves be associated with macular oedema, the possibility of confounding by underlying ocular disease deserves a little more emphasis.

**Response**: Thank you for your valuable feedback. The possibility that prostaglandin analogue eye drops can be a confounding factor is important point. We have added a note about it to the corresponding sections of the Discussion.

P. 12, L. 193 (Discussion)

Finally, reports involving prostaglandin analogue eye drops often co-existed with ocular diseases such as retinal disease and history of ocular surgery [21,38,41]. Therefore, prostaglandin analogue eye drops might be a confounding factor in the detection of signals for ME.

21. Muayad J, Mukhtar AO, Bordbar DD, Loya A, Alsoudi AF, Weng CY, et al. A large-scale cohort analysis of topical prostaglandin analog use and pseudophakic cystoid macular edema following cataract surgery. Ophthalmol Glaucoma. 2026;25: 276-281. doi: 10.1016/j.ogla.2025.12.010.

38. Moroi SE, Gottfredsdottir MS, Schteingart MT, Elner SG, Lee CM, Schertzer RM, et al. Cystoid macular edema associated with latanoprost therapy in a case series of patients with glaucoma and ocular hypertension. Ophthalmology. 1999;106: 1024-1029. doi: 10.1016/S0161-6420(99)00528-X.

41. Zhou Y, Bicket AK, Marwah S, Stein JD, Kishor KS, Pershing S, et al. Incidence of acute cystoid macular edema after starting a prostaglandin analog compared with other classes of glaucoma medications. Ophthalmol Glaucoma. 2025;8: 4-11.

**Responses to the comments by Reviewer 2:**

This is a concise and clinically relevant manuscript that effective connects the risk of Macular Edema (ME) with use of Paclitaxel in clinical trials. The author highlighted an important AE association; ME and PTX exposure while accounting for other variables. The reviewer thinks that the authors implied multivariable logistic regression while using the term multivariate logistic regression.

**Response**: Thank you for your valuable feedback. After referring to the literature as listed below, we totally agree that "multivariable logistic regression" is a more appropriate expression than "multivariate logistic regression,". Therefore, we have revised the manuscript accordingly (Lines 7, 14, 80, 106, 114, 118, 122, 126, and 169).

Bertha H, et al., Multivariate or Multivariable Regression?. Am J Public Health. 2013;103(1):39–40. doi: 10.2105/AJPH.2012.300897.
